# A novel MYB::PAIP1 oncogenic fusion in pediatric blastic plasmacytoid dendritic cell neoplasm (BPDCN) is dependent on BCL2 expression and is sensitive to venetoclax

**DOI:** 10.1002/hem3.1

**Published:** 2024-02-14

**Authors:** Hansen J. Kosasih, Gerry Healey, Margs S. Brennan, Stefan Bjelosevic, Teresa Sadras, Fatimah B. Jalud, Tasnia Ibnat, Ashley P. Ng, Chelsea Mayoh, Jie Mao, Gabor Tax, Louise E. A. Ludlow, Ricky W. Johnstone, Marco J. Herold, Seong L. Khaw, Charles E. de Bock, Paul G. Ekert

**Affiliations:** ^1^ Murdoch Children's Research Institute Parkville Victoria Australia; ^2^ Children's Cancer Institute, Lowy Cancer Research Centre UNSW Sydney Kensington New South Wales Australia; ^3^ The Walter and Eliza Hall Institute of Medical Research Parkville Victoria Australia; ^4^ Olivia Newton‐John Cancer Research Institute Heidelberg Victoria Australia; ^5^ Department of Medicine Huddinge, Centre for Haematology and Regenerative Medicine Karolinska Institutet Stockholm Sweden; ^6^ Peter MacCallum Cancer Centre Melbourne Victoria Australia; ^7^ The Sir Peter MacCallum Department of Oncology University of Melbourne Parkville Victoria Australia; ^8^ Department of Biology The University of Melbourne Parkville Victoria Australia; ^9^ School of Clinical Medicine, UNSW Medicine & Health UNSW Sydney Sydney New South Wales Australia; ^10^ Department of Paediatrics University of Melbourne Parkville Victoria Australia; ^11^ Department of Medical Biology University of Melbourne Parkville Victoria Australia; ^12^ School of Cancer Medicine La Trobe University Heidelberg Victoria Australia; ^13^ School of Women's and Children's Health UNSW Sydney Kensington New South Wales Australia

Blastic plasmacytoid dendritic cell neoplasm (BPDCN) is a rare and aggressive hematological malignancy with a median survival of 14 months (with a survival window of 6–28 months) from diagnosis.^1^ BPDCN is derived from the precursors of plasmacytoid dendritic cells^2^ and is recognized as an independent entity of myeloid neoplasms in the 2016 updated World Health Organization classification.^3^ Although BPDCN predominantly affects adults with a median age in the 70s, cases involving younger adults and children have been reported. Unique clinical presentations of BPDCN include skin infiltration, and at the molecular level, BPDCN is characterized by an overexpression of CD123 (IL‐3Rα).^4^ However, the pathogenesis of BPDCN remains incompletely understood. Here, we report a case of pediatric BPDCN with a novel MYB::PAIP1 rearrangement that is sufficient and necessary to drive leukemogenesis in vivo using a syngeneic murine transplant model.

The patient, a 10‐year‐old girl, presented with lethargy, generalized aches and a tender, violaceous skin lesion, and was found to have marked leukocytosis (white cell count 90 × 10^9^/L) comprised mainly of circulating blasts. Bone marrow aspirate demonstrated a predominant population (85%) of small‐to‐medium‐sized blasts without lineage‐defining features. Lumbar puncture demonstrated no cerebrospinal fluid pleocytosis, and no blasts on cytospin. The bone marrow blast immunophenotype was CD45^dim^, CD58+/CD56+/CD4+/CD123+. The karyotype was complex with loss of chr13, an unbalanced translocation between chr5q and chr6q, and derivative chr20 that matches previous aneuploidies (losses) of chromosomes 5, 6, and 13 reported in BPDCN.^5^ Microarray analysis showed deletion of chromosomes 6q23.3 (involving exons 9–16 of *MYB*), 7p12.2 (including *IKZF1*), and 9p21.3 (including *CKDN2A/2B*). Whilst BPDCN is classified as a myeloid neoplasm, there are features, such as *IKZF1* and *CDKN2A* deletions, that are shared with lymphoid neoplasms.^6^ The patient was treated following the Interfant‐06 protocol, selected based on inclusion on this protocol of myeloid‐directed elements on a predominantly acute lymphoblastic leukemia (ALL)‐based backbone, and previous reports of the superior efficacy of ALL‐based compared to acute myeloid leukemia‐based therapy in pediatric BPDCN.^7^ The patient had an excellent response to therapy and was in complete morphological remission at the end of induction therapy. She remained in remission and continued therapy following the same protocol until the end of the MARMA (methotrexate, ara‐C, 6‐mercaptopurine, PEG‐asparaginase) block. Due to the lack of a validated assay for quantifying minimal residual disease and thus confirming the depth of response and high risk of relapse associated with this disease, the patient underwent matched sibling donor hematopoietic stem cell transplant (HSCT), with busulfan, fludarabine, and thiotepa conditioning. She had a relatively uncomplicated posttransplant course and remains in complete remission with good donor chimerism 4 years post‐HSCT.

To further characterize this case at the molecular level, we performed RNA‐sequencing (RNA‐seq) on the diagnostic sample and used ALLSorts, a machine learning classifier of subtypes of B‐cell ALL (B‐ALL).^8^ This analysis revealed that this case of BPDCN was closely related to the BCL2/MYC subtype group, characterized by elevated *BCL2* and *MYC* expression resulting from translocations linking the immunoglobulin locus with either *BCL2* or *MYC* ^9^ (Supporting Information S2: Figure 1A). This is consistent with reported upregulation of *BCL2* and *MYC* expression in BPDCN.^10^, ^11^ Using Arriba,^12^ a fusion detection algorithm, a novel rearrangement between *MYB* (6q23.3) and *PAIP1* (5p12) was identified (Figure 1A and Supporting Information S4: Table 1). This novel rearrangement was confirmed by reverse‐transcriptase PCR across the breakpoint and Sanger sequencing (Figure 1A,B). The transcription factor MYB is essential for hematopoiesis and is a key regulator of MYC and BCL2.^13^ Different MYB rearrangements have previously been reported in 5/5 pediatric and 5/9 adult BPDCN cases.^14^ On the other hand, PAIP1 plays an important role in the regulation of protein translation by interacting with other co‐activators to promote messenger RNA circularization that initiates the translation,^15^ and its upregulation was reported in several types of solid cancers.^16^, ^17^ This novel rearrangement, between exon 8 of *MYB* and exon 3 of *PAIP1*, resulted in an in‐frame MYB::PAIP1 fusion, consisting of the MYB DNA‐binding domain, but lacking part of the MYB transcriptional activation domain and all of the negative regulatory domain, while also includes the MIF4G domain of PAIP1 and excludes some regions of PAIP1 that interact with translational initiation co‐activators (Figure 1C).

**Figure 1:**
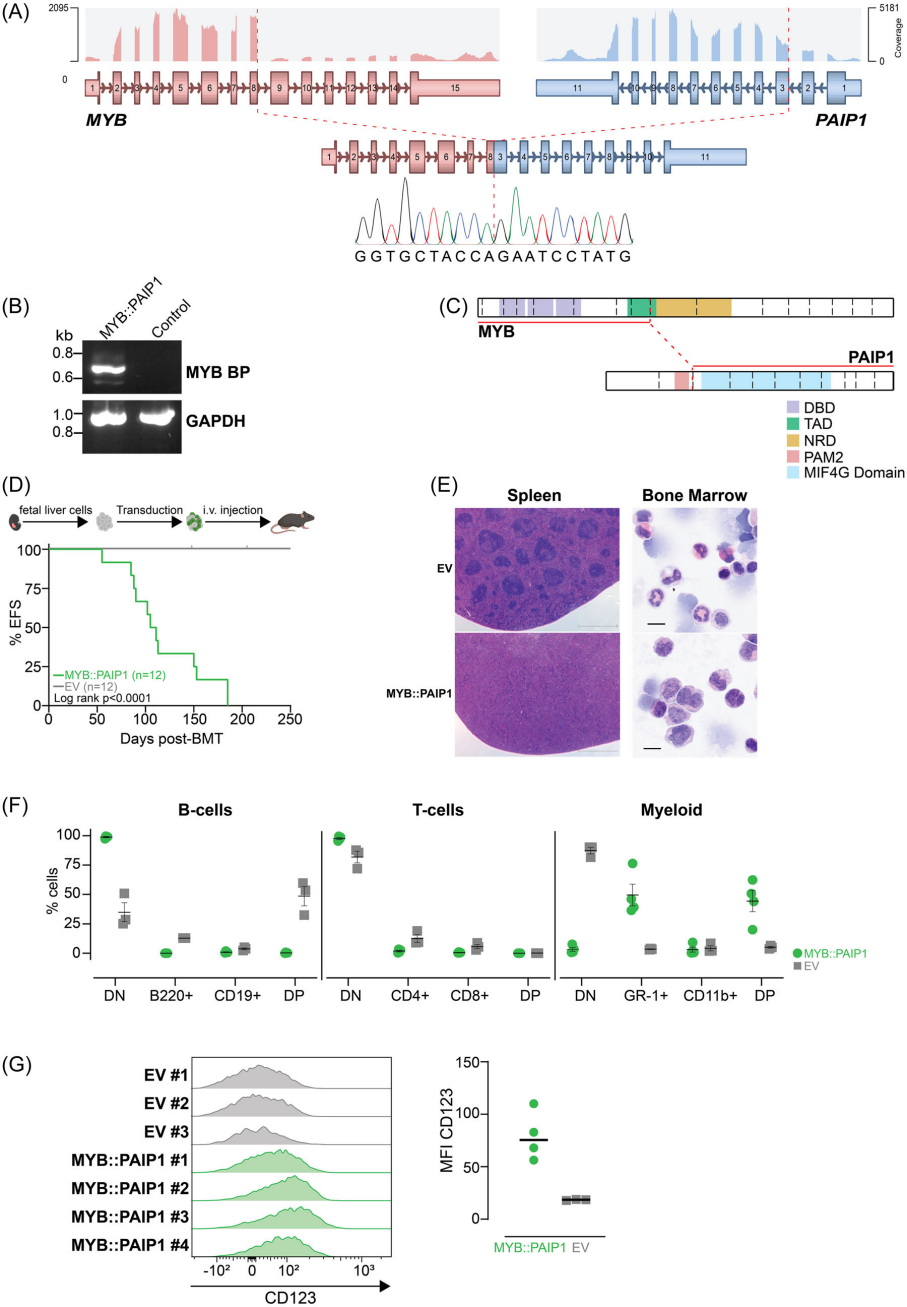
Novel MYB::PAIP1 fusion is sufficient and necessary to drive leukemia in vivo. (A) RNA‐sequencing analysis identified a novel rearrangement between *MYB* (6q23.3) and *PAIP1* (5p12), resulting in an in‐frame MYB::PAIP1 fusion. Image shown was modified from Arriba. Sanger sequencing result of the breakpoint region was shown. (B) Reverse transcription‐polymerase chain reaction of the patient with MYB::PAIP1 fusion and control patient sample. (C) Schematic illustrating the domains retained in the MYB and PAIP1 portions of the fusion. (D) Schematic showing the syngeneic mouse model used to determine the leukemogenic potential of MYB::PAIP1 fusion. Created with BioRender.com, and a Kaplan–Meier survival curve of mice receiving huMCL1 hematopoietic stem cells (HSCs) expressing the MYB::PAIP1 fusion (*n* = 12) or empty vector (EV) control (*n* = 12). There were two non‐leukemic related deaths in the EV control as indicated by ticks. (E) Representative images of hematoxylin and eosin staining of spleens (left panel) and bone marrow (BM) cytospins (right panel) from EV and MYB::PAIP1 mice. Scale bar of 200 µm on spleen histology and 10 µm on BM cytospins were shown. (F) Immunophenotyping analysis of the MYB::PAIP1 (four independent mice) and the EV cells (three independent mice). Lines represent mean values (±SEM). (G) Flow cytometry analysis of cell surface CD123 expression in MYB::PAIP1 (four independent mice) and the EV splenocytes (three independent mice) gated on single cells, viability, and GFP+, and the associated median fluorescence intensity (MFI) plot of this staining. Median values of MFI were shown as lines. BMT, bone marrow transplant; DBD, DNA‐binding domain; DN, double negatives; DP, double positives; EFS, event‐free survival; HSC, hematopoietic stem cell; MIF4G, middle domain of eukaryotic initiation factor 4G; NRD, negative regulatory domain; PAM2, PABPC1‐interacting motif‐2; TAD, transcription activation domain.

To investigate the oncogenic potential of this novel fusion, we cloned the fusion from cDNA derived from the tumor into a retroviral expression plasmid MSCV‐IRES‐GFP (MIG). The fusion was transduced into unsorted murine E14.5 fetal liver cells (FLCs) from humanized MCL1 (huMCL1) mice. The huMCL1 mouse strain (human *MCL1* gene knocked ‐into the murine *Mcl1* locus) is phenotypically indistinguishable from wild‐type C57BL/6 (WT).^18^ The strain was previously established to allow more precise testing of efficacy and tolerability of MCL1 inhibitors because of their higher affinity (up to sixfold) for human MCL1 compared to the mouse protein.^18^ Transduced cells were transplanted into sublethally γ‐irradiated syngeneic WT mice, while empty MIG vector‐bearing transduced cells (empty vector [EV]) were separately transplanted as controls, as previously described.^19^ Mice transplanted with cells expressing the MYB::PAIP1 fusion succumbed to a hematological malignancy with a median survival of 108 days in two independent cohorts, while EV‐transplanted mice did not develop the disease (*p* < 0.0001) (Figure 1D). MYB::PAIP1‐transplanted mice had enlarged spleens infiltrated with blast cells compared to the control mice (median 1.84% of body weight vs. 0.20%, *p* < 0.0001) (Figure 1E and Supporting Information S2: Figure 1B). Fusion‐bearing mice were anemic, and some had higher white blood count (WBC) compared to controls (median 16.57 × 10^9^ vs. 10.07 × 10^9^ cells/L, *p* = 0.065) (Supporting Information S2: Figure 1B). This observation was in accordance with the characteristics of BPDCN patients in whom anemia is often observed, while the WBCs are often within the normal range.^20^ There was a marked infiltration of the bone marrow with a homogenous population of monocytic blast cells in the fusion‐bearing mice (Figure 1E). Consistent with this observation, the MYB::PAIP1 fusion cells were exclusively GR1+CD11b− or GR1+CD11b+, and with relevance to BPCDN, also expressed higher levels of CD123 compared to the EV control cells (Figure 1F,G and Supporting Information S2: Figure 1C). To determine whether expression of the truncated form of MYB in the absence of the 3′ PAIP1 partner was sufficient to drive disease, an independent syngeneic mouse model experiment using FLCs from WT mice was undertaken comparing the leukemogenic potential of MYB portion present in the fusion (called MYB‐trunc) with MYB::PAIP1. Only mice bearing the MYB::PAIP1 fusion, and not the MYB‐trunc, succumbed to disease, with the MYB::PAIP1 disease also transplantable into secondary recipients resulting in reduced disease latency and demonstrating the presence of leukemic stem cells (*p* = 0.0177) (Supporting Information S3: Figure 1D). This finding indicates that PAIP1 portion is important to the function of this novel fusion. We speculate the MIF4G domain might recruit co‐factors into a transcriptional complex that leads to deregulated gene expression.

We next assessed the transcriptomic profile of the MYB::PAIP cells and the EV cells harvested from the bone marrow (BM) of the huMCL1 cohorts. The cells were sorted for B220−/CD19− to compare a similar population of cells. Differential gene expression analyses revealed alterations in expression of genes (*Bcl2, Il3ra* that encodes for CD123, *Sox4, Igll1, Egfr, Irf4, Cxcr3*) that typically dysregulated in BPDCN (Figure 2A).^21^ Upregulation of *Myc* (Log FC 1.60, *p* < 0.001) and *Bcl2* (Log FC 1.73, *p* < 0.001) in the fusion‐bearing cells were in concordance with the ALLSorts classification of the diagnostic patient sample (Figure 2A). Gene set enrichment analysis (GSEA) was enriched for MYB target genes, suggesting that MYB‐dependent targets are critical to this model system (Figure 2B) and consistent with MYB‐activating events seen in younger cases of BPDCN.^14^ GSEA also showed positive correlations with early to late progenitors of hematopoiesis, and negative correlation with mature hematopoietic cells in MYB::PAIP1 cells (Supporting Information S3: Figure 1E), implying the expression of this fusion blocked normal hematopoietic differentiation, in accordance with the observed morphology of the cells (Figure 1E). Importantly, the GSEA also showed positive correlations with MYC targets and E2F targets in the fusion‐bearing cells, consistent with oncogene‐associated signatures in BPDCN patients (Figure 2B and Supporting Information S3: Figure 1F).^21^

**Figure 2:**
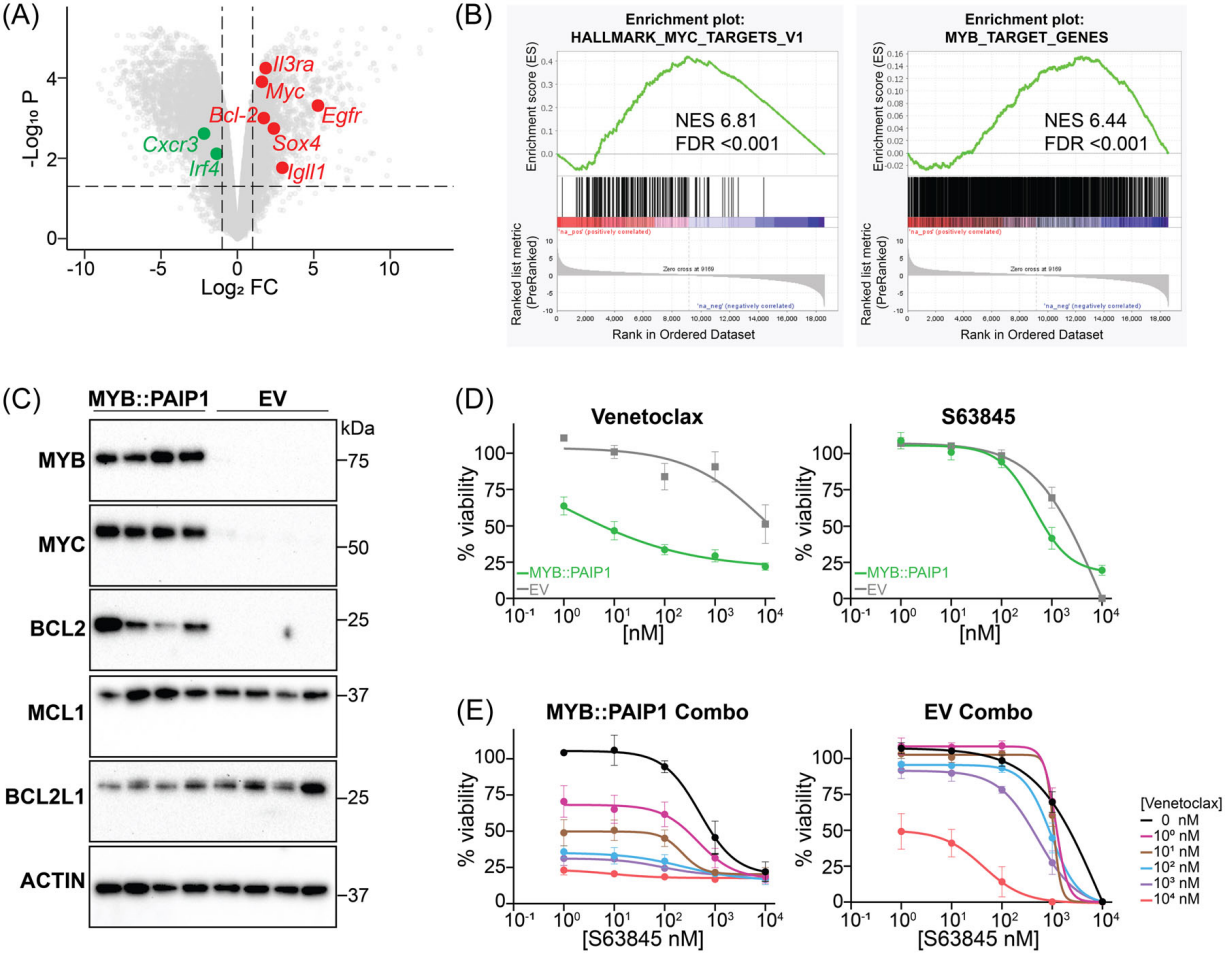
MYB::PAIP1 cells are dependent on BCL2 expression and are sensitive to venetoclax. (A) Volcano plot of differentially expressed genes between MYB:PAIP1 (*n* = 3) and empty vector (EV) control cells (*n* = 3). (B) Gene set enrichment analysis (GSEA) from the same RNA‐sequencing experiment shows significant enrichment for hallmark MYC and MYB targets in MYB::PAIP1 cells. (C) Western blot analysis of MYB::PAIP1 and EV‐bearing bone marrow cells for MYB, MYC, BCL2, MCL1, BCL2L1, and ACTIN loading control. (D) Dose response for MYB:PAIP1 cells treated with the BH3‐mimetic venetoclax, and the MCL1 inhibitor S63845 after 24 h exposure. All treatments were done in technical duplicates, with three biological replicates. The cell viability data was normalized against the dimethyl sulfoxide‐treated cells. Mean (±SEM) was shown. (E) Dose response for the combination of venetoclax and S63845 in MYB::PAIP1‐ and EV‐bearing cells. Mean (±SEM) was shown. FDR, false discovery rate; NES, normalized enrichment score.

We then validated the upregulation of *Myc* and *Bcl2* at the protein level, with MYB::PAIP1 cells having higher expression of MYC and BCL2 compared to the EV‐bearing cells. There was no significant difference in the expression of the other antiapoptotic proteins (BCL2L1, MCL1) (Figure 2C). On this basis, we hypothesized that the fusion‐bearing cells would be uniquely sensitive to the BCL2 inhibitor venetoclax but not the MCL1 inhibitor S63845. The use of huMCL1 model allowed us to accurately assess the sensitivity of the cells to S63845, as we have previously shown.^18^ The MYB::PAIP1 fusion‐bearing cells were more sensitive to venetoclax (half‐maximal inhibitory concentration [IC_50_] 1.032 nM) compared to S63845 (IC_50_ 456.7 nM) (Figure 2D). The combination of S63845 and venetoclax was not synergistic in MYB::PAIP1 cells upon 24 h exposure, suggesting that these cells were acutely sensitive to BCL2 inhibition ex vivo (Figure 2E and Supporting Information S3: Figure 1G). It is possible that the fusion‐bearing cells could become sensitive to MCL1 or BCL2L1 inhibition after selection in venetoclax. Taken together, these data support the leukemogenic potential of the MYB::PAIP1 fusion through increased BCL2 expression that can be acutely exploited therapeutically via the BH3‐mimetic venetoclax in this murine model system.

There is currently no standardized therapy for pediatric BPDCN patients, with most patients receiving high‐risk ALL treatment protocols. Tagraxofusp, a CD123‐directed antibody conjugate, was recently approved for the treatment of BPDCN, but serious adverse events were common in adults and the clinical efficacy was disappointing.^22^ Our data suggest that in MYB‐fusion‐positive BPDCN cases, venetoclax may represent a possible targeted therapy since the fusion drives BCL2 expression, at least in our model. Indeed, the successful use of venetoclax has been reported in adult cases of BPCDN^23^, ^24^ and is also being investigated in a current clinical trial combining venetoclax with chemotherapy (HCVAD) and CD123‐targeted therapy in adults.^25^ One case report also mentioned the use of venetoclax combined with hyper‐CVAD, in an 11‐year‐old boy who had relapsed BPDCN, where complete response was achieved.^26^ Our study provides a strong molecular link and rationale for further investigations of the clinical use of venetoclax in MYB‐fusion‐driven BPCDN that has the potential to improve the outcome of pediatric BPDCN patients.

## AUTHOR CONTRIBUTIONS

Hansen J. Kosasih and Paul G. Ekert conceived and designed the study. Hansen J. Kosasih performed all the experiments, analyzed the data, and wrote the manuscript. Gerry Healey, Margs S. Brennan, and Stefan Bjelosevic assisted in vivo experiments. Teresa Sadras, Fatimah B. Jalud, and Tasnia Ibnat performed the cytospins and imaged the tissue histologies. Ashley P. Ng imaged the cytospins and provided the assessments on the cell morphology. Jie Mao and Gabor Tax assisted in the analysis of the drug synergism experiment. Chelsea Mayoh and Jie Mao assisted in GSEA analysis. Ricky W. Johnstone and Marco J. Herold contributed to the conception and conduct of in vivo experiments. Louise E. A. Ludlow and Seong L. Khaw contributed to the identification of eligible samples and the collation and presentation of clinical data. Paul G. Ekert and Charles E. de Bock assisted in the analysis of experimental data and the writing of the manuscript. All authors contributed to the manuscript preparation.

## CONFLICT OF INTEREST STATEMENT

Paul G. Ekert and Seong L. Khaw are recipients of a share in milestone and royalty payments paid to the Walter and Eliza Hall Institute of Medical Research for the development of venetoclax.

## FUNDING

This work is supported by National Health and Medical Research Council Grants (1140626 to P. G. E.), and a SCOR Grant (7015‐18 to P. G. E. and S. L. K.) from the Leukemia and Lymphoma Society. S. L. K. and H. J. K. were supported by the Children's Cancer Foundation and the Department of Health and Human Services through the Victorian Cancer Agency (Project 134). M. S. B. is supported by the Cancer Council Victoria Postdoctoral Fellowship and Swedish Cancer Society (21 0355 PT). T. S. is supported by a Gilead Research Scholars Award and funding from the Kid's Cancer Project Mid‐Career Fellowship. A. P. N. is supported by a grant‐in‐aid from Cancer Council Victoria, National Stem Cell Foundation of Australia Metcalf Prize for Stem Cell Research, Alessandra's Fight Against Leukaemia (GoFundMe), and a Leukaemia Foundation Breakthrough Fellowship. R. J. W. is supported by a project grant from Cancer Council Victoria, an investigator grant from the NHMRC, and a grant from the Kids' Cancer Project. We also acknowledge the support of Perpetual Trustees and the Samuel Nissen Foundation.

## Supporting information

Supporting information.

Supporting information.

Supporting information.

Supporting information.

Supporting information.

## Data Availability

The data that supports the findings of this study are available in the Supporting Information of this article. The breakpoint sequence and genomic locations of the novel MYB::PAIP1 fusion are provided in Supplementary Table 1 as part of the Arriba output. Detailed information can be obtained from the corresponding author upon reasonable request.
