# Supplementary material for: A novel MYB::PAIP1 oncogenic fusion in pediatric blastic plasmacytoid dendritic cell neoplasm (BPDCN) is dependent on BCL2 expression and is sensitive to venetoclax
Source: Hemasphere. 2024 Feb 14;8(2):e1. doi: 10.1002/hem3.1 (PMC10878182; doi:10.1002/hem3.1)
Supplement: Supplementary file 1 — Supporting information. [file HEM3-8-e1-s004.docx]

**Supplementary Methods**

**RNA isolation, library preparation, and fusion detection of the patient sample**

Informed consent for collection and use of the patient sample was obtained by Children’s Cancer Centre Tissue Bank according to the Royal Children’s Hospital Human Ethics Committee guidelines (HREC 34127). Patient RNA was extracted from a peripheral blood mononuclear cells using the AllPrep DNA/RNA/miRNA Universal Kit (Qiagen). High quality RNA was subjected to a library preparation using TruSeq stranded mRNA (Illumina). The library was sequenced on the Illumina NovaSeq 6000 using 2x150bp paired-end reads at 80M reads depth. The .fastq file was analyzed using a multi-step pipeline that contains FastQC to check the quality of the .fastq file^1^; adapter trimming using trimmomatic^2^; alignment to human genome (GRCh38) using STAR^3^; samtools to sort, merge, and index the .bam file^4^; and finally counting the number of reads map to genes using featureCounts^5^. The output .bam file was inputted to Arriba, a fusion detection algorithm to find any potential oncogenic fusions using the suggested parameters for fusion detection^6^. The gene counts matrix was used as an input for ALLSorts to determine whether the patient was closely related to any subtypes of B-Cell Acute Lymphoblastic Leukemia (B-ALL)^7^.

**Cloning of the fusion**

RNA from the patient was reverse-transcribed to cDNA using SuperScript III Reverse Transcriptase (Invitrogen). The expression of the fusion was initially confirmed by breakpoint PCR using the primers set that amplify the breakpoint region of the fusion: Fwd primer: GCCAGCCCACTGTTAACAAC and Rev primer: TCTGCTCTTGTAACCTGTCCA. The full-length fusion was amplified using the following primers set: Fwd primer: TAAGTTATAAGCCATGGCCCGAAGACCCCG and Rev primer: CTTACTCGAGTTACTGTTTTCGCTTACGCTCTGATTCCAA. The truncate version of MYB was amplified using the same forward primer as the full-length fusion, but with Rev primer: CTTACTCGAGTTATGGTAGCACCTGCTGTCCTTTTAG. All PCR amplification reactions were done using Q5 High-Fidelity DNA Polymerase (NEB). Both the full-length fusion and the truncate version were cloned into a constitutive retroviral expression plasmid MSCV-IRES-GFP (MIG).

**Retroviral transduction and in vivo transplantation**

The MYB constructs were co-transfected with the appropriate retroviral packaging constructs (mouse ENV and retroviral gagpol) into 293T cells, either by using Effectene Transfection Reagent (Qiagen) or home-made Calcium Phosphate/HEPES Buffered Saline mix to produce viral supernatants. Unsorted fetal liver cells (FLCs) harvested from murine E14.5 (either from humanised MCL-1 mice or C57BL/6 mice) were cultured in α-minimum essential medium (α-MEM) (Invitrogen) supplemented with 10% foetal calf serum (FCS), 2 mM L-glutamine, murine stem cell factor (SCF) (100 ng/ml) (PeproTech), IL-6 (10 ng/ml), thrombopoietin (50 ng/ml), and FMS-related tyrosine kinase 3 (FLT) (10 ng/ml) (all produced at WEHI) and transduced with viral supernatants using the RetroNectin protocol (double spin infection)^8^. The transduced cells were injected into sub-lethally γ-irradiated syngeneic wild-type C57BL/6 (WT) mice. Mice were monitored for signs of leukemia (weight loss, enlarged spleen, lethargy, and hunched posture) and sacrificed at the first sign of disease. Upon sacrifice, the GFP expression in BM, spleen, and liver were assessed to confirm the engraftment of the cells containing the fusion. Harvested bone marrow cells and splenocytes were frozen in 90% FCS + 10% Dimethyl Sulfoxide (DMSO). Kaplan-Meier survival curves were plotted and compared using Log-rank (Mantel-Cox) test. All in vivo experiments were conducted in accordance with the Walter and Eliza Hall Institute (WEHI) Animal Ethics Committee (AEC) and Peter MacCallum Cancer Centre AEC guidelines.

**Cytospins and Histology**

Bone marrow samples were cytospun on a Shandon Cytospin 3, fixed in methanol, and stained for haematoxylin & eosin (H&E) using a Leica Autostainer. Bone marrow cell morphology was examined under oil immersion microscopy x 60 objective lens. Whole spleens were fixed in 10% formalin, dehydrated in ethanol and embedded in paraffin. Tissues were sectioned to 3 µm thickness and stained with H&E. Images were acquired with an EVOS m5000 microscope.

**Immunophenotyping of the ex-vivo cells**

Frozen bone marrow (BM) cells or splenocytes were thawed quickly in 37°C water bath, washed in Iscove’s Modified Dulbecco’s Medium (IMDM) supplemented with 10% FCS, and counted. Five hundred thousand cells were collected and blocked in 49µl staining buffer (Phosphate Buffered Saline (PBS) containing 2% FCS) and 1µl TruStain FcX (anti-mouse CD16/32) antibody (BioLegend) for 30 minutes at 4°C. The cells were washed in PBS and resuspended for staining in 33µl staining buffer, 10µl BD Horizon Brilliant Stain Buffer, and the following antibody cocktail: anti-mouse CD45R/B220 (RA3-6B2) APC, anti-mouse CD19 (1D3) BV786, anti-mouse CD11b (M1/70) BUV395, anti-mouse Ly-6G and Ly-6C (Gr-1) (RB6-8C5) BUV737, anti-mouse CD4 (GK1.5) BUV496, anti-mouse CD8 (53-6.7) BV650, and anti-mouse CD123 (5B11) BV711, all were used at 1:50 dilution, for 30 minutes at 4°C. After a PBS wash, cells were resuspended in the staining buffer for analysis on BD LSRFortessa X-20 (BD Biosciences). The cells were gated on alive (by DAPI exclusion) and single cells prior to quantifying stained cells on the relevant fluorochrome channels. All antibodies were obtained from BD Biosciences.

**RNA-Seq analysis of the ex-vivo cells**

Mouse BM cells from the huMCL-1 cohorts were thawed, stained with anti-mouse CD45R/B220 APC cl. RA3-6B2 and anti-mouse CD19 BV786 cl. 1D3, and sorted on the BD Influx Cell Sorter to select for B220- and CD19- cells. RNA from the sorted cells were isolated using PureLink RNA mini-kit (ThermoFisher) and subjected to library preparation using Illumina Ribo-Zero Plus. The library was sequenced paired-end (2x150bp) at a sequencing depth of 50M reads. The data were processed as described above, with the exception of aligning to mouse reference genome mm10. Differential Expression analysis of the was performed in R using the edgeR and limma Bioconductor packages^9,10^. The volcano plot was generated using EnhancedVolcano^11^. Gene Set Enrichment Analysis (GSEA) was done using GSEA app ^12,13^. Briefly, the ranked list was created from the DGE result by using the LogFC as the metric and ranked by the adjusted P-Value. This list was feed into the GSEA app: Run GSEAPreranked. The parameters were set as follows: Gene sets database: mh.all.v2023.2.Mm.symbols.gmt; Number of permutations: 1000; Collapse/Remap to gene symbols: No_Collapse; Enrichment statistic: classic; Max size: 500; Min size: 15

**Western blot analysis**

Up to 2x10^6^ BM cells from the mice in the huMCL-1 cohorts were directly lysed by boiling them for 10 minutes in PBS containing SDS sample buffer, 0.1M dithiothreitol (DTT) and 5% β-mercaptoethanol in a total volume that gave a final concentration of 20,000 cells/µl. An equivalent of 200,000 cells were loaded on 4-15% Mini-PROTEAN TGX precast protein gels (Bio-Rad). Protein gels were transferred onto Immobilon-E PVDF membranes using the wet transfer protocol and probed with the appropriate antibodies after blocking with 5% skim milk according to the standard Western blot protocol. The following primary antibodies were used: MYB (CST #12319), MYC (CST #9402), MCL1 (CST #94296), BCL2 (CST #3498), BCL2L1 (CST #2764), and ACTIN (CST #58169). The following secondary antibodies were used: HRP-conjugated donkey anti-rabbit IgG or anti-mouse IgG (Cytiva). Immobilon Forte Western HRP substrate (Merck) was used as the chemiluminescent HRP detection reagent.

**Drug Assay of the ex-vivo cells**

BM cells from the huMCL-1 cohorts were thawed, counted, and seeded on 96-well plate at 50,000 cells/well in 50µl IMDM + 10% FCS + 10ng/ml mIL-3 (Peprotech). Venetoclax or S63845 (both from Selleckchem) was separately diluted to the desired concentration in IMDM + 10% FCS + 10ng/ml mIL-3. For a combination treatment, 25µl of each drug was added to the appropriate well to achieve a final concentration of 1nM, 10nM, 100nM, 1µM, and 10µM for each drug. For a single treatment, 25µl of each drug was added to the appropriate well, together with 25µl IMDM + 10%FCS + 10ng/ml mIL-3 containing DMSO (the diluent of the drug stock). For untreated well, 50µl IMDM + 10%FCS + 10ng/ml mIL-3 containing DMSO was added to the well. The final seeding density of the BM cells on each well was 500,000 cells/ml. Following 24-hour drug exposure at 37°C/5%CO2, viability was determined by DAPI exclusion of GFP+ cells using BD LSRFortessa X-20. All treatments were done in technical duplicates, with 3 biological replicates. The cell viability data was normalised against the DMSO treated cells. For the drug synergism analysis, the nature of drug-drug interactions was established by calculating Bliss independency scores.

1. Andrews S. FastQC: A Quality Control Tool for High Throughput Sequence Data [Online]. 2010

2. Bolger AM, Lohse M, Usadel B. Trimmomatic: a flexible trimmer for Illumina sequence data. *Bioinformatics*. Aug 1 2014;30(15):2114-20. doi:10.1093/bioinformatics/btu170

3. Dobin A, Davis CA, Schlesinger F, et al. STAR: ultrafast universal RNA-seq aligner. *Bioinformatics*. Jan 1 2013;29(1):15-21. doi:10.1093/bioinformatics/bts635

4. Li H, Handsaker B, Wysoker A, et al. The Sequence Alignment/Map format and SAMtools. *Bioinformatics*. Aug 15 2009;25(16):2078-9. doi:10.1093/bioinformatics/btp352

5. Liao Y, Smyth GK, Shi W. featureCounts: an efficient general purpose program for assigning sequence reads to genomic features. *Bioinformatics*. Apr 1 2014;30(7):923-30. doi:10.1093/bioinformatics/btt656

6. Uhrig S, Ellermann J, Walther T, et al. Accurate and efficient detection of gene fusions from RNA sequencing data. *Genome Res*. Mar 2021;31(3):448-460. doi:10.1101/gr.257246.119

7. Schmidt B, Brown LM, Ryland GL, et al. ALLSorts: an RNA-Seq subtype classifier for B-cell acute lymphoblastic leukemia. *Blood Adv*. Jul 26 2022;6(14):4093-4097. doi:10.1182/bloodadvances.2021005894

8. Brumatti G, Ma C, Lalaoui N, et al. The caspase-8 inhibitor emricasan combines with the SMAC mimetic birinapant to induce necroptosis and treat acute myeloid leukemia. *Sci Transl Med*. May 18 2016;8(339):339ra69. doi:10.1126/scitranslmed.aad3099

9. Robinson MD, McCarthy DJ, Smyth GK. edgeR: a Bioconductor package for differential expression analysis of digital gene expression data. *Bioinformatics*. Jan 1 2010;26(1):139-40. doi:10.1093/bioinformatics/btp616

10. Ritchie ME, Phipson B, Wu D, et al. limma powers differential expression analyses for RNA-sequencing and microarray studies. *Nucleic Acids Res*. Apr 20 2015;43(7):e47. doi:10.1093/nar/gkv007

11. Blighe K, Rana S, Lewis M. EnhancedVolcano: Publication-ready volcano plots with enhanced colouring and labeling. 2018.

12. Mootha VK, Lindgren CM, Eriksson KF, et al. PGC-1alpha-responsive genes involved in oxidative phosphorylation are coordinately downregulated in human diabetes. *Nat Genet*. Jul 2003;34(3):267-73. doi:10.1038/ng1180

13. Subramanian A, Tamayo P, Mootha VK, et al. Gene set enrichment analysis: a knowledge-based approach for interpreting genome-wide expression profiles. *Proc Natl Acad Sci U S A*. Oct 25 2005;102(43):15545-50. doi:10.1073/pnas.0506580102
