# Supplementary figures and images for: A novel MYB::PAIP1 oncogenic fusion in pediatric blastic plasmacytoid dendritic cell neoplasm (BPDCN) is dependent on BCL2 expression and is sensitive to venetoclax
Source: Hemasphere. 2024 Feb 14;8(2):e1. doi: 10.1002/hem3.1 (PMC10878182; doi:10.1002/hem3.1)

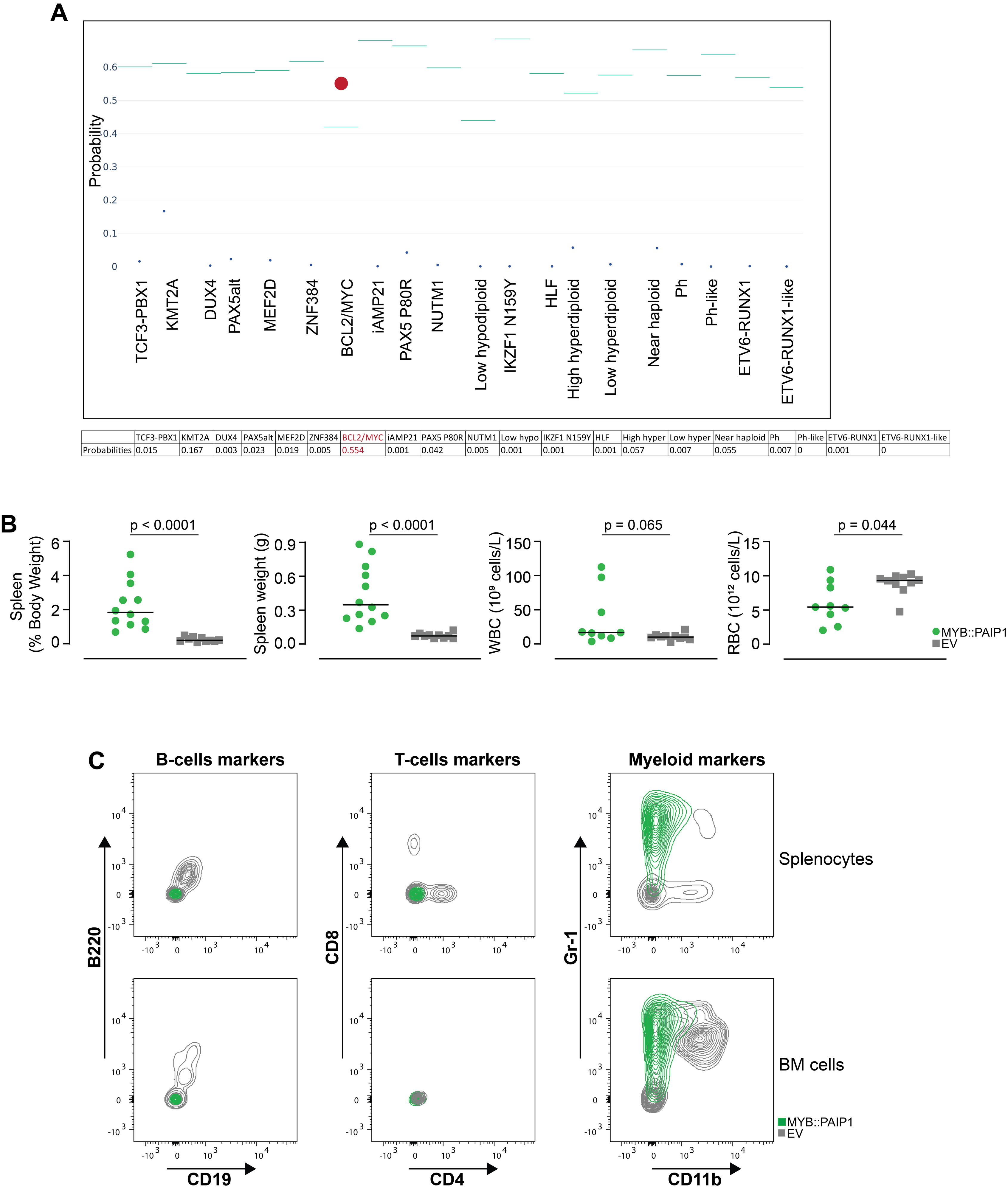

Supplement: Supplementary file 2 — Supporting information. [file HEM3-8-e1-s001.jpg]

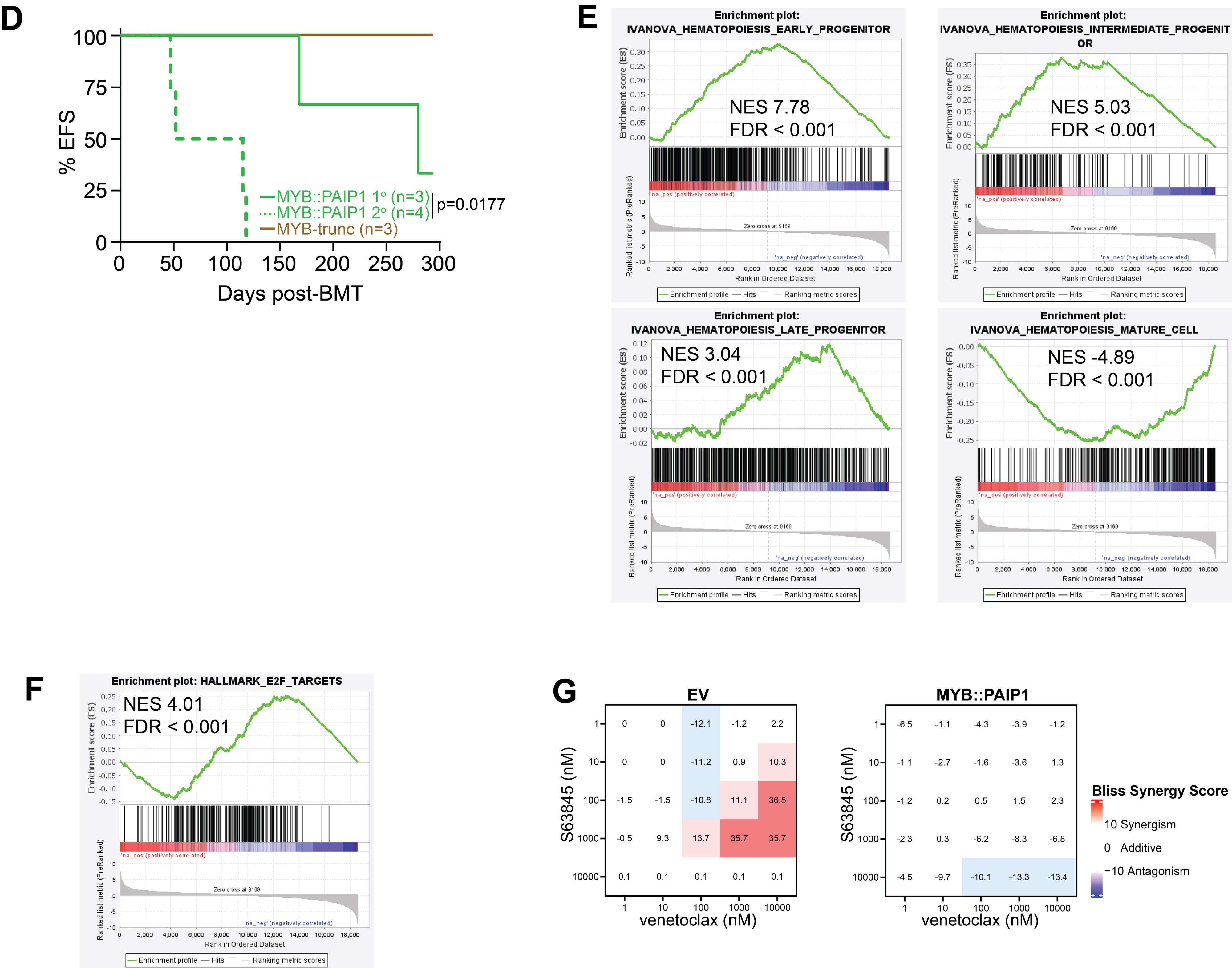

Supplement: Supplementary file 3 — Supporting information. [file HEM3-8-e1-s005.jpg]
